# Cognitive abilities in a sample of young Swedish children

**DOI:** 10.3389/fpsyg.2024.1398398

**Published:** 2024-12-03

**Authors:** Ingela Clausén Gull, Johanna Stålnacke, Lilianne Eninger, Laura Ferrer-Wreder, Kyle Eichas

**Affiliations:** ^1^Department of Psychology, Stockholm University, Stockholm, Sweden; ^2^Department of Psychological Sciences, Tarleton State University, Stephenville, TX, United States

**Keywords:** cognitive abilities, executive functioning, preliteracy, social emotional competence, Sweden, children, confirmatory factor analysis, structural equation modeling

## Abstract

Cognitive abilities are closely related to social emotional competences (SEC). These abilities are important foundations in order to adapt to school, interact with peers and adults, as well as to navigate the wider socio-cultural context in which one develops. Further, young children are also acquiring and deepening their language and preliteracy skills which are important for later academic learning. Central to cognitive abilities are the processes that enable deliberate and goal-oriented actions, which fall under the conceptual umbrella of executive functions (EFs). In this study, we applied a conceptually broad perspective to examine cognitive abilities, preliteracy and SEC in preschool aged children. Children were participants in an intervention trial of the preschool edition of Promoting Alternative Thinking Strategies (PATHS^®^) conducted in preschools located in three municipalities within a large city in Sweden. Pre-test data were used to examine cognitive abilities and SEC in this sample of Swedish 4 to 5-year-old children (*N* = 247). We first performed an exploratory factor analysis including the wide range of examined abilities, and found that measures of abilities typically viewed as SEC, did not group with measures of preliteracy skills and abilities typically considered as EFs. Second, we performed confirmatory factor analyses on remaining relevant indicators of cognitive abilities, which indicated a two-factor model best fit the data, with one factor involving inhibitory control and one factor involving more complex and high-demanding skills (working memory, cognitive flexibility, and preliteracy skills). Results indicated that more complex EFs and preliteracy skills were closely linked, and can be differentiated from inhibitory control, already in the preschool years. Findings also point to the importance of including a broad range of cognitive abilities (e.g., pre-literacy skills) in order to gain a nuanced description of possible interrelations between cognitive and social emotional development. Furthermore, this study contributes to the theoretical discussion on EF structure during childhood, and provides a sound empirical rationale for the further development of early interventions that consider young children’s executive functions and preliteracy skills.

## 1 Introduction

During the first years of a child’s life, tremendous development takes place in communication through spoken and written language, and in understanding of the world. Central to these changes are the development of cognitive abilities and social emotional competence (SEC; Blair and Raver, 2015). Cognitive abilities are, in this article, broadly conceptualized as the jointly nested cognitive processes that allow us to, for example, perceive and attend to our surroundings, remember relevant information, and solve problems in everyday situations. Further, central to cognitive abilities are processes that enable deliberate and goal-oriented actions and that fall under the conceptual umbrella of executive functions (EFs). EFs are generally considered to include inhibitory control (i.e., self-direction of individuals’ internal life that helps them to act effectively in the world), working memory (i.e., maintaining and using information) and cognitive flexibility (i.e., shifting to meet changes and taking in standpoints of others) (Baggetta and Alexander, 2016; Diamond, 2013).

Social emotional competence can be expressed by doing well and thriving in school, learning how to interact with peers and adults, and navigating the socio-cultural context in which one develops (Hecht and Shin, 2015; Weissberg et al., 2015). Within the Collaborative for Academic and Social and Emotional Learning’s [CASEL] framework (Collaborative for Academic, Social, and Emotional Learning [CASEL], 2024; Weissberg et al., 2015), development of SEC involves integration of affective, behavioral and cognitive abilities across five core competences: self-awareness; self-management; responsible decision making; relationship skills; and social awareness. The specific facets of SEC examined in this study included emotional knowledge (i.e., an indicator of self-awareness), social problem solving (i.e., relationship skills), and theory of mind [i.e., social awareness (Collaborative for Academic, Social, and Emotional Learning [CASEL], 2024; Weissberg et al., 2015)]. Further, the EFs examined in this study are typically viewed as facets of SEC within the self-management area of competence (Collaborative for Academic, Social, and Emotional Learning [CASEL], 2024; Weissberg et al., 2015). Thus, within the CASEL framework, cognitive abilities and SEC are intertwined, and in this study, we examined the latent structure of these constructs from this conceptually broad perspective in a sample of young Swedish children.

### 1.1 Relevant prior research and theories

The REDI intervention paired both social emotional learning (SEL) and literacy training in a preschool setting in the U.S. (Bierman et al., 2008a). Studies of SEL (Collaborative for Academic, Social, and Emotional Learning [CASEL], 2024) indicate that interventions, like the REDI trial (Bierman et al., 2008a), can improve children’s SEC, as well as strengthen children’s cognitive abilities, including EFs and preliteracy skills (Bierman et al., 2008b; Sasser et al., 2017). Other results have shown that children with more room to grow in EFs, particularly benefit from preschool SEL interventions that also include a language-literacy focus (Bierman et al., 2008b; Sasser et al., 2017). Thus, from the SEL intervention field, there is evidence for some reciprocal development among cognitive abilities and related SECs during the preschool years, and this nexus of core competencies is an important area to support in a diversity of ways.

Indeed, cognitive abilities and SEC are important foundations for later well-being (Bierman et al., 2021; Jones et al., 2015; Weissberg et al., 2015), and are vital assets to rely upon when meeting academic tasks and challenges (Serpell and Esposito, 2016; Weissberg et al., 2015; Zelazo and Carlson, 2020). In young children, cognitive abilities develop rapidly (Garon et al., 2008; Reilly et al., 2022) and around 4–5 years of age, children appear to deepen and integrate key abilities underpinning school readiness (Bierman et al., 2008a,b; Blair and Raver, 2015; Welsh et al., 2010). In addition, EF shows robust associations with language and emergent literacy (Hooper et al., 2020; Welsh et al., 2010) as well as with SEC (Bierman et al., 2008b; Ștefan et al., 2022). These are competencies that facilitate children’s abilities to adapt and thrive in school and are fundamental to interacting with peers and adults. Acquiring language and preliteracy skills in preschool are also important for later writing and reading and, consequently, academic learning (Bierman et al., 2008b; Hooper et al., 2020; Shaul and Schwartz, 2013).

#### 1.1.1 Structure and differentiation of executive functions (EFs)

In an influential study with young adults by Miyake et al. (2000), EFs were defined as a set of three differentiable, yet highly interrelated, cognitive components, namely inhibition, updating/working memory, and shifting/cognitive flexibility. This conceptualization was later updated with a complementary model that included a common EF factor (corresponding with inhibition), one updating/working memory-specific factor and one shifting-specific factor (Miyake and Friedman, 2012). The structure of EF core components proposed by Miyake and Friedman (2012) have been replicated in many adult samples (e.g., Baggetta and Alexander, 2016; Karr et al., 2018). However, the development of EFs are not yet fully documented when considering how EFs work in different developmental periods and in light of how contexts of development vary across the globe. Another factor of importance, in this field of study, includes a careful consideration and comparison across varying global contexts of development to determine how measurement tools perform psychometrically as indices of the constructs in question (e.g., Thomas et al., 2021).

Although additional research is needed, there is an evidence base regarding the precursors of EF (e.g., Garon et al., 2008; Hendry et al., 2016), as well as the developmental trajectories of the EF components (Diamond, 2013; Garon et al., 2008; Reilly et al., 2022), and findings show that EF components (i.e., inhibition, working memory, cognitive flexibility) seem to be less differentiated in younger relative to older children (Baggetta and Alexander, 2016; Hendry et al., 2016; Karr et al., 2018; Reilly et al., 2022). Garon et al. (2008) delineated the development of EF, pointing to inhibition as the earliest building block, from which rudimentary working memory function develops, and this then sets the stage for more complex EF components such as cognitive flexibility. These EF components are also important for semantic learning (Filipe et al., 2023) and adaptive behavior (Baggetta and Alexander, 2016; Racz et al., 2017; Serpell and Esposito, 2016).

In previous research, efforts have been made to conceptualize EF in young children through delineating constructs with the use of confirmatory factor analysis (CFA) of children’s performance on various tasks. Baggetta and Alexander (2016) performed a systematic review of the conceptualization and operationalization of EFs which were examined in 106 empirical studies published since 2008, covering ages from infants to older adults. The authors concluded that there seems to be a conceptual convergence, with EFs appearing to be unidimensional early in life but becoming increasingly multidimensional as individuals enter into adulthood.

Although a general pattern over developmental time has been identified, there remains a lack of widespread agreement as to when the move from unity to diversity in EFs takes place among children and youth who have grown up in different life circumstances (e.g., Karr et al., 2018; Michel and Bimmüller, 2023; Monette et al., 2015). Recent CFA studies of children’s EF have shown support for single (e.g., Hughes et al., 2009; Reilly et al., 2022) as well as two-factor models (e.g., Miller et al., 2012; Monette et al., 2015). For example, Miller et al. (2012) used CFA to describe the factor structure of a battery of EF tests with 129 preschool children living in Canada (age 3–5 years old). Results indicated that a two-factor model best fit the data, i.e., latent factors reflecting inhibition and working memory. Further, in another Canadian sample of kindergartners (*N* = 272; age five to 6 years old), Monette et al. (2015) reported a two-factor model including one factor of inhibition and one factor reflecting working memory-flexibility. Moreover, a two-factor model with factors for inhibition and shifting/updating also best fit the data in a German sample of 175 children, 4–7 years of age (Michel and Bimmüller, 2023), and in a sample of 3–4 year old preschool children (*N* = 117) in Italy (Scionti and Marzocchi, 2021). There is also evidence that factors may be identifiable already in the first 3 years of life. In a review, Hendry et al. (2016) presented a conceptual model suggesting that emergent EF could be conceptualized as two latent factors, inhibitory control skills (impulse control) and cognitive flexibility (in which updating and shifting are closely intertwined). Evidence for a three-factor model of EF in young children is scarce, although investigated in several studies (e.g., Miller et al., 2012; Monette et al., 2015; Karr et al., 2018). However, some measurement limitations may obscure the picture, in that in some studies, the full range of indicators of three EF core components was lacking, which then reduces the chances to obtain a model with more than one factor (Monette et al., 2015).

A systematic review performed by Karr et al. (2018) of studies using CFAs on EF (46 samples, *N* = 9,756) showed that the most frequently accepted models varied by age. Studies including samples of school-aged children (six to 12 years), adolescents/adults (13–17 years/18–59 years) and older adults (older than 60 years) mainly presented three-factor models that most often included inhibition, updating/working memory, and cognitive flexibility/shifting factors (Karr et al., 2018). For preschool samples (children younger than 6 years old), both one-factor and two-factor models were most descriptive of these data across studies (Karr et al., 2018). However, no study identified a specific, distinct factor for cognitive flexibility/shifting (Karr et al., 2018). The nine studies with the preschool samples were conducted in Canada, the U.S. and Italy. In this review, there were no studies from Sweden with preschool children (Karr et al., 2018).

In summary, the current research literature supports the idea that EFs can be less differentiated in young children relative to older children, adolescents and adults (Baggetta and Alexander, 2016; Ibbotson, 2023). Thus, the empirical particulars and timing of these changes in diverse cohorts of young children needs further investigation, particularly when considering the diversity in child development as well as living conditions among young children from a variety of cultures and contexts across the globe.

#### 1.1.2 Why examine cognitive abilities, including preliteracy skills?

Several studies have broadened our conception of the intersections between cognitive and social emotional development in children by linking aspects of cognitive abilities to SECs, as these abilities are important for school readiness as well as the other social and cultural tasks that children navigate (Baggetta and Alexander, 2016). For example, one such cognitive ability is emergent literacy, which includes knowledge about letters and words (Bierman et al., 2008b; Shaul and Schwartz, 2013). In the aforementioned REDI trial, EFs (i.e., working memory, inhibitory control, and set shifting skills) assessed at the beginning of the intervention trial, at base line, significantly predicted emergent literacy at the end of school year (Bierman et al., 2008b; Welsh et al., 2010). Several other studies have shown EFs to be predictive of language skills (Filipe et al., 2023; Tonér, 2021; Tonér and Gerholm, 2021) and emerging literacy (Shaul and Schwartz, 2013; Welsh et al., 2010). Thus, in the present study, a conceptually broad perspective (e.g., skills of importance for academic learning and SECs) was used as a starting point to examine cognitive abilities and SEC in preschool aged children.

### 1.2 Cognitive abilities — as conceptualized and measured in this study

In line with previous conceptualizations of EF (Diamond, 2013; Garon et al., 2008; Hendry et al., 2016), the present study included measures of inhibitory control, working memory and cognitive flexibility in an attempt to cover the breadth of EFs as would be developmentally expected and fitting for use with this study sample of young children. Inhibitory control was indexed with four child tasks [Knock and Tap (NEPSY; Korkman et al., 1998, 2000); Statue (NEPSY; Korkman et al., 1998, 2000); Day/night Stroop-like task (Berlin and Bohlin, 2002); and Go/no-go (Berlin and Bohlin, 2002; Brocki et al., 2007)], with each task aiming to capture slightly different aspects of the overall construct of inhibitory control, such as simpler forms, like inhibiting a dominant response, or more complex forms, like interference control (see Diamond, 2013; Nigg, 2017).

In turn, working memory, which involves the ability to maintain and manipulate information, (Diamond, 2013) was, in this study, captured using a word span task (Tillman et al., 2008). Working memory is essential to a number of abilities, for example, being able to carry out a task according to given instructions (Best and Miller, 2010; Diamond, 2013; Hendry et al., 2016), making sense of spoken or written language (Diamond, 2013; Filipe et al., 2023; Tonér and Gerholm, 2021), and being able to retell a story (Tonér and Gerholm, 2021).

Cognitive flexibility was measured, in the present study, using a set-shifting task similar to the hearts and flowers task (Davidson et al., 2006). This entails remembering two conflicting instructions and switching between them, which requires considerable cognitive resources (Diamond, 2013; Garcia and Dick, 2013), and likely requiring a combination of inhibitory control and working memory (see Brocki and Tillman, 2014). Preliteracy skills, referring to the early skills needed for later reading and writing (Hooper et al., 2020), were also examined in this study, with a focus on letter knowledge (Johansson, 2009) and object recognition (Samuelsson et al., 2005; Wagner et al., 1999).

Further, examined abilities in this study included skills needed for social interaction and regulation of thoughts and emotions, abilities that can be viewed as consistent with CASEL’s SEC model (Collaborative for Academic, Social, and Emotional Learning [CASEL], 2024; Weissberg et al., 2015). Apart from the facet of *self-management*, which was reflected in the EFs described earlier in this section, *self-awareness* was examined in terms of emotional knowledge (ACES; Schultz et al., 2004), i.e., recognizing and labeling one’s own and others’ emotional expressions. *Social awareness* was measured by the ability to understand false-beliefs and differing perspectives, i.e., theory of mind (False belief; Hughes et al., 2005; Sullivan et al., 1994). *Relationship skills* were studied through successful social problem solving, also requiring the ability to understand another person’s perspective (CST; Denham et al., 1994).

### 1.3 Study context

Promoting Alternative Thinking Strategies (PATHS^©^) is a universal SEL intervention, that is consistent with the CASEL framework and can be argued to have some elements of language-literacy that are infused within lessons designed to promote SEC (Domitrovich et al., 2007). In Sweden, a randomized controlled trial (RCT) of PATHS was conducted in preschools located in three municipalities within a large urban city (Eninger et al., 2021).

In the wider PATHS intervention trial, approximately 43% children attended preschools in economically disadvantaged neighborhoods and 57% children attended preschools in economically advantaged neighborhoods (Kapetanovic et al., 2022). Preschool neighborhood level advantage/disadvantage was determined based on registry data for all residents living in the postal code in which children’s preschools were located during the intervention trial (i.e., all residents’ monthly income before taxes based on registry data). The average resident income by postal code for all preschool postal codes was then compared against the entire region average during the time period of the intervention trial to determine neighborhood preschool contexts that were above the regional average income (advantaged) or below the regional average income (disadvantaged; Kapetanovic et al., 2022).

It should be noted that the wider PATHS intervention trial happened across two waves [Wave 1: April 2014–June 2015 (pre to post-test) and Wave 2: April 2015–June 2016 (pre to post-test)]. As schools were recruited into the study, they were block randomized to an intervention or wait-list control condition. The implementation of the PATHS curriculum was for approximately one academic school year in the immediate intervention condition schools. Presently, there is only pre to post test data for this PATHS trial. A long term follow up study of immediate intervention participants is currently underway.

In the present study, the pre-test data, before the PATHS intervention was conducted, are used for the analyses that examine cognitive abilities and SEC in this sample of Swedish four to five-year-old children. In addition to the child tasks reported in this study, the assessment battery for the overall project included observations of children’s prosocial skills when in a structured play situation and teacher ratings of participating children’s: Prosocial/communication skills, emotional self-regulation, academic skills, social cooperation, interaction, and independence, as well as indicators of children’s social withdrawal, anxiety/somatic symptoms, aggression, inattention and hyperactivity/impulsivity (for more about the trial see Eninger et al., 2021).

The wider Nordic welfare model is essential to understand the Swedish preschool context (Ferrer-Wreder et al., 2020). This model is characterized by ideals of working toward a democratic and egalitarian view of society, and aspiring to high standards of well-being and quality of life for everyone including children (Ferrer-Wreder et al., 2020). Nordic Early Childhood Education and Care (ECEC) policies have been developed in the context of an integrated model that combine the interests of the labor market, and families and children, with the aim to support parents so they have an equal opportunity to participate in the labor market as well as home life (Alexiadou et al., 2024; Karila, 2012). Swedish preschools are mostly government-subsidized, and open for all children from 1–5 years of age. Preschool enrollment in Sweden is generally high, with 79% of one to three-year-old’s and 95% of four-and five-year-old’s attending preschool (Swedish National Agency for Education: Skolverket, 2022). In Sweden, as well as in the other Nordic countries, many preschool teachers are well educated and practice is guided by a national curriculum for preschool education, although variations in these practices across schools and classrooms exist (Alexiadou et al., 2024; Coelho et al., 2021; Karila, 2012).

In the Swedish national curriculum for preschools, early academic learning as well as children’s SEC are central in that children’s play is considered an important foundation for development, learning and well-being, but also for promoting, for example, language, inter-personal relations and social problem-solving (Swedish National Agency for Education: Skolverket, 2018). Pedagogically, this entails supporting children in their learning and creativity so that they can develop a positive self-conception and confidence in their own abilities, as well as acquire valuable knowledge and insights through their own agency. Psychologically, this entails supporting their developing emotional knowledge and regulation, empathy, communication, and problem-solving skills, all of which are essential to engaging in and contributing to relationships. Furthermore, the preschool curriculum emphasizes language development (Swedish National Agency for Education: Skolverket, 2018), and there is a long tradition of shared book reading in Swedish preschools (Hofslundsengen et al., 2020). A study by Hofslundsengen et al. (2020) found that, in 131 Nordic classrooms (ages 3–6 years old for a majority of the classrooms), books were common and accessible for children, and use of children’s books was a main gateway to literacy.

### 1.4 Study aim and contributions

The factor structure of a broadly defined set of cognitive abilities, which include indices of EFs, preliteracy skills, as well as other indicators of SECs (i.e., 12 constructs) were investigated in this study. The research questions had a two-step approach. An exploratory factor analysis (EFA for RQ1) examined the overall shared variance among all 12 study constructs (e.g., EFs, preliteracy, emotional knowledge, social problem solving, theory of mind, and general non-verbal ability). An EFA with all 12 constructs was first conducted due to the lack of any prior published Swedish study with this age group of children, and with such as wide array of constructs which touched on several aspects of the CASEL model as well as aspects of language development. Informed by the results from RQ1, a conceptually more focused CFA approach was then used with the relevant indicators of cognitive abilities that were empirically (in this sample) and theoretically associated (testing construct validity and scale reliability).

*RQ1*: From a wide conceptual standpoint, and by using EFA, how do the 12 measures of cognitive abilities group (i.e., associate with one another) in an empirical and conceptual manner in this sample?

*RQ2*: Are the indicators of cognitive abilities identified in RQ1 best described with a one-factor, two-factor model, or a bifactor model, as tested by CFA analyses?

This study has several contributions in that it examines a broad view of children’s cognitive and social emotional development, and tests the expectation from the research literature and theory that there could be empirical synergies between abilities in a sample of young children. Further, to our knowledge, no prior Swedish (or Nordic) empirical study has yet examined such as wide array of child tasks that measure cognitive abilities, which include social and emotional competences, and has used two factor analytic approaches (EFA and CFA).

## 2 Materials and methods

### 2.1 Participants

Participants were 247 children aged 4–5 years old (*M* = 58.6 months, *SD* = 5.5 months; 48.6% identified as girls). Children were participants in an intervention trial of the preschool edition of Promoting Alternative Thinking Strategies (PATHS^®^) in Sweden. This was a randomized controlled trial with implementation at the preschool level. There were intervention and waitlist control group schools and pre and post-test assessments conducted in children’s preschools. Details and main outcome from the intervention study are reported by Eninger et al. (2021). In brief, 26 preschools from three municipalities in a large Swedish city participated in the study. The present study focuses on data from the child tasks at pretest, prior to any participation in the PATHS intervention, and encompasses all participating children who were 4–5 years old, from both the intervention and control preschools across two data collections waves.

### 2.2 Measures

The conceptual connections between the tasks described below, cognitive abilities, relevant overarching constructs and core competences within CASEL’s SEC Model are summarized in Table 1.

**Table 1:** Tasks, cognitive abilities, relevant overarching constructs and core competences within CASEL’s SEC model.

Task	Cognitive abilities	Construct/core competence
Knock and Tap (K&T)	Inhibitory control; control of motor response, within task interference	Executive function/Self-management
Statue (Stat)	Inhibitory control; control of motor response, distraction outside task	Executive function/Self-management
Opposites (Opp)	Inhibitory control; interference control of verbal response, within task interference	Executive function/Self-management
Go/No-go (GnG)	Inhibitory control; interference control of pre-potent response	Executive function/Self-management
Word Span (WS)	Working memory	Executive function/Self-management
Apples and Pears (A&P)	Cognitive flexibility	Executive function/Self-management
Block Design (Bd)	General non-verbal ability	General cognitive ability
Rapid Automatized Naming (RAN)	Preliteracy skills; early literacy, vocabulary	School oriented general cognitive ability
Letter Knowledge (L)	Preliteracy skills; early literacy, crystalized knowledge	School oriented general cognitive ability
The Assessment of Children’s Emotional Skills (ACES)	Emotional knowledge	Social emotional competence/Self-awareness
False Belief (Fb)	Theory of mind	Social emotional competence/Social awareness
Challenging Situations Task (CST)	Emotional awareness and social problem solving	Social emotional competence/Self-awareness and Relationship skills

Inhibitory control measures: Knock and Tap (K&T), Statue (Stat), Opposites (Opp), and Go/No-go (GnG). Working memory measure: Word Span (WS). Cognitive flexibility measure: Apples and Pears (A&P). General non-verbal ability: Block design (Bd). Preliteracy skill measures: Rapid Automatized Naming (RAN) and Letter Knowledge (L). Emotional knowledge measures: Assessment of children’s emotional skills (ACES) and False belief (Fb). Social problem-solving measure: Challenging situations task (CST).

#### 2.2.1 Inhibitory control

##### 2.2.1.1 Knock and Tap (K&T)

This task is designed to assess inhibitory control and involves interference control of a motor response. From a wider standpoint, this is an indicator of EF which is within the self-management facet of the Collaborative for Academic, Social, and Emotional Learning [CASEL] (2024) model. For this task, the child is instructed to perform specific hand movements in response to the experimenter’s hand movements. For instance, to knock on the table in response to the experimenter placing her or his palm on the table (NEPSY; Korkman et al., 1998, 2000). The task score is the number of correct child responses, a maximum of 30, with a higher score indicating better inhibitory control. For this sample, split-half reliability for the Knock and Tap task was positive and significant at 0.22, *p* = 0.002 (Eninger et al., 2021).

##### 2.2.1.2 Statue (Stat)

As the K&T task, this task also measures inhibitory control and consists of interference control of a motor response [i.e., an EF indictor, self-management facet of the Collaborative for Academic, Social, and Emotional Learning [CASEL] (2024) model]. In this case, the child is asked to stand still and quiet with eyes closed for 75 s. The experimenter distracts the child by, e.g., dropping a pencil, knock the table at specific intervals (NEPSY; Korkman et al., 1998, 2000). The task score consists of the sum of the number of 5 s intervals that the pose is held by the child, with higher scores indicating better inhibitory control. For this task, test–retest reliability, based on a sample of American children was 0.50 for three to four-year-old children, and 0.75 for children five to six-year-old’s (NEPSY; Korkman et al., 1998).

##### 2.2.1.3 Opposites (Opp)

This task provides an indicator of inhibitory control that involves interference control [i.e., an EF indicator, self-management facet of the Collaborative for Academic, Social, and Emotional Learning [CASEL] (2024) model]. Using a computer tablet, the child is presented with different pictures that represent opposites, i.e., up/down and large/small. For each picture, the child is asked to say the opposite word, for example, to say “large” when presented with a picture of a small present, thus inhibiting the meaning of the picture shown. This is an adapted version (Berlin and Bohlin, 2002) of the Stroop-like Day-Night task (Gerstadt et al., 1994). The task score is the sum of the number of the correct child responses. Scores can range from zero to 48, and a higher score indicates better inhibitory control. Thorell et al. (2006) used the Day-Night task (performed on a computer instead of a computer tablet) with a test–retest reliability of 0.84 (*p* < 0.0001) for 22 Swedish children from 4–5 years old, tested 2 weeks apart.

##### 2.2.1.4 Go/No-go (GnG)

This task is designed to measure interference control [i.e., an EF indicator, self-management facet of the Collaborative for Academic, Social, and Emotional Learning [CASEL] (2024) model]. The task involves showing children shapes (squares or circles) in red or blue presented on a computer tablet. In the first block the child is asked to respond to all blue shapes, and in the second block to respond to all squares (Berlin and Bohlin, 2002; Brocki et al., 2007). In a similar task, performed on a computer and not a computer tablet, Thorell (2007) obtained a test–retest reliability of 0.62 for a sample of six-year-old Swedish children (*n* = 145). The task score is the sum of the number of commission errors made. A maximum score of 18 is possible, and higher scores implies weaker interference control.

#### 2.2.2 Working memory

##### 2.2.2.1 Word span (WS)

This task aims to provide an indicator of working memory [i.e., an EF indicator, self-management facet of the Collaborative for Academic, Social, and Emotional Learning [CASEL] (2024) model]. In this task, the child is orally presented a series of one or two-syllable words, and the child is instructed to remember the words and repeat them in the same order after each trial. The trials increase from two to six words, with two list series in each trial (Tillman et al., 2008). The task score is the sum of the number of correctly remembered word-pairs worked in the same order as presented to the child, and for this sample Cronbach’s alpha was 0.63 (Eninger et al., 2021). Scores can range from zero to 30, with a higher number indicating better working memory.

#### 2.2.3 Cognitive flexibility

##### 2.2.3.1 Apples and Pears (A&P)

This task provides an indicator of cognitive flexibility [i.e., an EF indicator, self-management facet of the Collaborative for Academic, Social, and Emotional Learning [CASEL] (2024) model]. The task involves the presentation of an apple or a pear on either side of the screen of a computer tablet. When the apple appears, the child is asked to press on the same side that the apple appears. When presented with a pear, the child is asked to press on the opposite side of the tablet. In the mixed trial, apples and pears appear randomly within the same series, making this trial cognitively more demanding. This task is adapted from Hearts and Flowers task (Davidson et al., 2006), and task score is the sum of the number of correct responses in the mixed condition. Scores can range from zero to a maximum of 20, with higher scores indicating better cognitive flexibility.

#### 2.2.4 General non-verbal ability

##### 2.2.4.1 Block design (Bd)

This task is designed to provide an indicator of general non-verbal ability. In this task, the child is asked to build blocks from a model or picture. The test is from the standardized Wechsler preschool intelligence test, WPPSI-III, Swedish version, and was administered according to the test manual instructions (Wechsler, 2002, 2005). The task score is the sum of the number of points for the child’s performance in this task, the maximum score is 40. A higher score on this task is meant to reflect greater general non-verbal ability. The administration and scoring of this task were initially unclear for the administrators, which lowers the quality of the data.

#### 2.2.5 Preliteracy skills

##### 2.2.5.1 Rapid Automatized Naming (RAN)

The Rapid Automatized Naming test (Samuelsson et al., 2005; Wagner et al., 1999) measures early literacy skills and vocabulary (i.e., a school oriented general cognitive ability). For this task, children are presented pictures of common objects and is requested to name the objects as quickly as they can. The task score is the number of seconds that a child takes to name all 20 objects. A higher score indicates poorer performance on this task. Internal consistency of this task has been reported as 0.71 (Samuelsson et al., 2005).

##### 2.2.5.2 Letter Knowledge (L)

The Letter Knowledge test (Johansson, 2009) provides a measure of early literacy skills and provides an indicator of crystalized knowledge (i.e., a school oriented general cognitive ability). In this task, all upper-case letters are presented to the child in a random order. If the child performs well on this condition, the lower-case letters are presented. The child is asked to name each letter he/she recognizes. As very few children passed on to identifying the lower-case letters, only the upper-case condition was used in the present study analyses. The task score is the sum of the correctly named upper-case letters, with a maximum score of 29. A higher score indicates greater early literacy skills and crystalized knowledge.

#### 2.2.6 Emotional knowledge

##### 2.2.6.1 Assessment of Children’ Emotional Skills (ACES)

This task is designed to measure children’s emotional knowledge [i.e., social emotional competence, facet of self-awareness in the Collaborative for Academic, Social, and Emotional Learning [CASEL] (2024) model]. In this task, the child is asked to label emotions (angry, scared, sad, and happy) from photos of children’s faces (Schultz et al., 2004). Of the 14 photos that were shown to the child, 10 of the photos had a single clear emotion displayed, based on pilot tests of the faces with Swedish children. The clear emotional faces were used for the task score, which was the sum of the correctly labeled faces. Cronbach’s alpha for this sample was 0.87 (Eninger et al., 2021). Scores can range from zero to 10, with higher scores indicative of greater emotional knowledge.

#### 2.2.7 False belief

##### 2.2.7.1 False belief (Fb)

This task provides an indicator of theory of mind [i.e., social emotional competence, facet of social awareness in the Collaborative for Academic, Social, and Emotional Learning [CASEL] (2024) model]. For this task, two short vignettes are presented to a child, one vignette at a time. A control question is part of the procedure for this task and is asked to a child to ensure comprehension of the task, prior to a question related to theory of mind (Hughes et al., 2005; Sullivan et al., 1994). The task score is the sum of the correct theory of mind responses with a maximum score of two.

#### 2.2.8 Social problem solving

##### 2.2.8.1 Challenging situations task (CST)

This task aims to tap emotional awareness and social problem-solving skills [Self-awareness and Relationship skills in the Collaborative for Academic, Social, and Emotional Learning [CASEL] (2024) model]. The task consists of four vignettes of a social situation that involves a social challenge or conflict that is presented to the child. For example, another child takes your favorite toy. The child is asked how they would feel if they were in the same situation as the child in the vignette. Children are also asked what they think they would do in the hypothetical situation in the vignette (Denham et al., 1994). Open-ended responses were coded by two researchers based on labeling of emotions and type of solution. Inter-rater reliability, based on ratings from either of the main coders vs. the ratings of a third coder who recoded a random subset of the data (about 10%), was calculated using Intraclass Correlations Coefficients (ICC) for each type of response, i.e., Competent (ICC = 0.77), Aggressive (ICC = 0.97), or Inept (ICC = 0.73), and Labeled emotions (emotional awareness; ICC = 0.91). The primary outcome measure used for the study is the competent solutions. The task score is the sum of competent solutions generated by children across vignettes. Higher scores indicate better competent social problem solving.

### 2.3 Procedure

A total of 25 trained research assistants took part in four periods of data collection (pretest and posttest, across two waves), of which eight research assistants were active at each data collection period. The same test protocol and order were used, and the tasks were chosen to index key facets of EF and school readiness, as well as designed to be sensitive indicators of possible changes as a result of PATHS participation (Eninger et al., 2021). In total, 12 tasks were administered individually to each child by a pair of research assistants, and the research assistants also rated child’s task orientation during the assessment. Most children completed the assessment in two sessions over the course of assessment across 1–2 days. Several children did not complete all tasks, and some chose to decline further participation. When children declined further participation, this was noted by the research assistants, and the assessment ended.

The PATHS project in Sweden was approved by a regional ethics board (dnr: 2012/1741–31/5). School personnel provided assent for study involvement. Parents provided written consent for their child’s study participation, and children provided verbal assent prior to their study participation. Children were informed that if they wished to stop the assessment, they could do so at any time.

### 2.4 Data analyses

**Figure 1:**
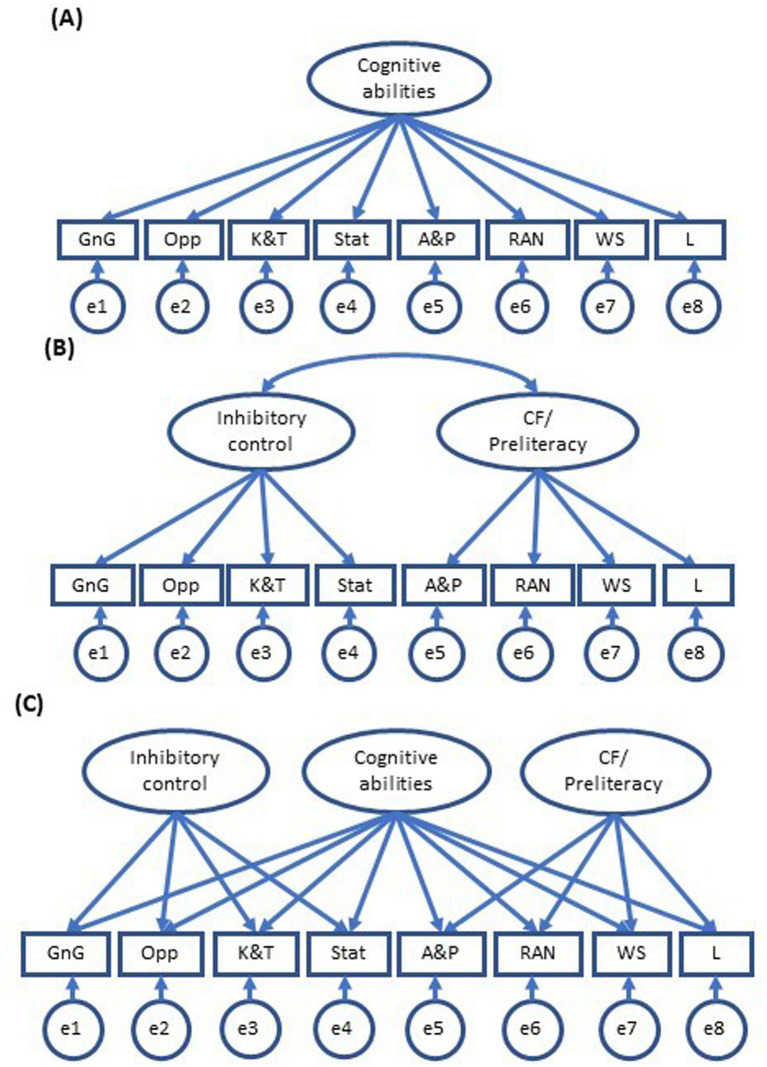
Competing models. **(A)** Unidimensional model, **(B)** two-factor model, **(C)** bifactor model observed variables: GnG, Go/No-go; Opp, Opposites; K&T, Knock and Tap; Stat, Statue; A&P, Apples and Pears; RAN, Rapid Naming; WS, Word Span; and L, Letters. For simplicity, factor loadings are not presented.

The analyses were conducted in steps that are consistent with RQ1 and RQ2, and begin from a wide conceptual standpoint with all 12 constructs and then based on the EFA, move into a more theory based and focused approach with a series of CFAs. Before the research questions were addressed, descriptive analyses were performed in SPSS 27 and 29 for PC (IBM Corp., Armonk, NY, United States). Associations between scales were examined with correlational analyses at the observed scale level (Pearson’s *r*; see Supplementary Table S1). Next, an EFA was conducted with all 12 child tasks (scale scores for the tasks: K&T, Stat, Opp, GnG, WS, A&P, Bd, RAN, L, ACES, Fb, and CST). Then, based on the results of the EFA, eight scales were retained for further analysis (i.e., K&T, Stat, Opp, GnG, WS, A&P, RAN, and L). Then, we examined RQ2 with a series of theoretically and empirically-based CFAs that tested the relative utility of a one, two, and bifactor model (see Figure 1).

The EFA and CFAs were performed with the use of structural equation modeling using Mplus 8.4 (Muthén and Muthén, 1998–2017). For all factor analyses, we used a robust maximum likelihood estimator for model estimation and a Huber-White adjustment to the standard errors (via Mplus’s TYPE = COMPLEX feature) to account for nesting of children within their preschools. Missing data ranged from 2.1 to 16.2% across the study constructs. We used full information maximum likelihood to estimate missing data.

For the factor analyses, goodness-of-fit measures were: (a) a Chi-square test as measurement of the discrepancy between the model-implied and sample moments; a non-significant chi-square (*p* ≥ 0.05) implies that exact fit between the model and the data cannot be ruled out; (b) the Comparative Fit Index (CFI) and the Tucker–Lewis Index (TLI), both being incremental fit indices comparing the specified model to a more parsimonious independence model. Values over 0.95, preferably over 0.97, respectively, are indicative of a good model fit (Hu and Bentler, 1999); (c) the Root Mean Square Error of Approximation (RMSEA) is a measure of absolute fit that calculates the discrepancy between model-predicted and observed correlations per degree of freedom. A value less than 0.08 indicates a good model fit (Hu and Bentler, 1999). Confidence intervals (90%) of RMSEA are provided as well as a *p*-value associated with testing if RMSEA ≤0.05; (d) the Standardized Root Mean Square Residual (SRMR), is an absolute fit measure and a standardized measure for the evaluation of model residuals. It reflects the average residual in the residual correlation matrix. An SRMR value of 0 indicates perfect fit, a value below 0.05 is considered to indicate good fit (Hu and Bentler, 1999), and (e) the Bayesian Information Criteria (BIC), an information theory-based index that can be used to compare different models. Lower values indicate better relative fit. The Chi-square difference test used the Satorra-Bentler scaled chi-square (Satorra and Bentler, 2010).

## 3 Results

Descriptive statistics for all 12 tasks are presented in Table 2. Consistent with RQ1, we conducted an EFA with the 12 tasks and examined models with one to four factors. The three-and four-factor models did not converge. However, as shown in Table 3, both the one-and two-factor models met all criteria for good model fit. The comparison of the one-and two-factor models revealed that the one-factor model had better relative fit than the two-factor model, BIC difference = 34.32. Moreover, the nested chi square difference test was not significant at an alpha level of 0.05, *χ*^2^ diff (11) = 17.55, *p* = 0.09.

**Table 2:** Mean and standard deviations, range, skewness and kurtosis for all measures.

Task	*n*	Mean (sd)	Range	Skewness (std error)	Kurtosis (std error)
Inhibitory control					
Go/No-go (GnG)	237	2,49 (3.18)	0 to 17	2.24 (0.16)	5.91 (0.32)
Knock and Tap (K&T)	226	24.33 (6.15)	1 to 30	−1.46 (0.16)	1.64 (0.32)
Opposites (Opp)	215	29.55 (12.64)	1 to 47	−0.54 (0.17)	−0.89 (0.33)
Statue (Stat)	207	23.13 (7.05)	2 to 30	−1.11 (0.17)	0.37 (0.34)
Cognitive flexibility/preliteracy					
Apples and Pears (A&P)	230	8.88 (4.38)	0 to 20	0.26 (0.16)	−0.44 (0.32)
Rapid Naming (RAN)	242	29,81(6,81)	16 to 55	0.89 (0.16)	1,13 (0.31)
Word Span (WS)	240	11,08 (4.25)	2 to 23	−0.002 (0.16)	−0.37 (0.31)
Letter Knowledge (L)	238	8.82 (9.07)	0 to 29	0.95 (0.16)	−0.46 (0.31)
Other baseline child tasks (included in exploratory factor analysis only)					
False belief	240	1.19 (0.77)	0 to 2	−0.34 (0.16)	−1.23 (0.31)
ACES^1^	245	7.05 (1,68)	1 to10	−1.00 (0.16)	1.65 (0.31)
CST^2^ Competent responses	244	2.40 (2.24)	0 to 8	0.74 (0.16)	−0.46 (0.31)
Block design (Bd)	238	20.89 (5.10)	6 to 34	−0.56 (0.16)	0.09 (0.31)

Inhibitory control measures: Go/No-go (GnG), Knock and Tap (K&T), Opposites (Opp), and Statue (Stat). Cognitive flexibility/Preliteracy measures: Apples and Pears (A&P), Rapid Automatized Naming (RAN), Word Span (WS) and Letter Knowledge (L). Tasks only included in exploratory factor analysis: False belief (Fb), ^1^Assessment of children’s emotional skills (ACES), ^2^Challenging situations task (CST) scale score on competent responses, Block design (Bd).

**Table 3:** Fit indices and model comparisons for competing models.

	Model fit								Model comparisons	
	df	Chi-square	RMSEA	SRMR	CFI	TLI	BIC		Vs. model	Difference test*
Model		*χ*^2^-statistic (*P*-value)	Estimate (Prob <=0.05)						(Δdf)	Δ*χ*^2^ (*P*-value)
Exploratory factor analyses										
1. 1-factor	54	62.885 (0.191)	0.026 (0.953)	0.046	0.974	0.968	15686.664		–	–
2. 2-factor	43	42.693 (0.485)	0.000 (0.985)	0.035	1.000	1,000	15720.984		1. (11)	17.55 (0.093)
3. 3-factor	–	–	–	–	–	–	–		–	–
4. 4-factor	–	–	–	–	–	–	–		–	–
Confirmatory factor analyses										
5. 1-factor	20	34.387 (0.024)	0.054 (0.382)	0.046	0.951	0.932	11655.508		–	–
6. 2-factor	19	20.214 (0.382)	0.016 (0.884)	0.035	0.996	0.994	11646.024		5. (1)	9.98 (0.002)
7. Bifactor	12	9.900 (0.625)	0.000 (0.926)	0.020	1.000	1.000	11673.226		6. (7)	9.59 (0.213)

*Satorra–Bentler scaled Chi-square difference test for MLR-estimated models; T-statistic. Df, degrees of freedom; RMSEA, root square error of approximation; CFI, comparative fit index; TLI, Tucker–Lewis Index; SRMR, standardized root mean square residual; BIC, Bayesian Information Criteria.

Based on the results from the RQ1, i.e., the EFA results, and guided by theoretical considerations, eight tasks were retained for further analysis in relation to RQ2. Four of these measures reflect inhibitory control (e.g., GnG, K&T, Opp, and Stat), whereas the other four measures reflect cognitive flexibility, working memory and preliteracy skills (A&P, RAN, WS, and L). The four tasks that were not chosen for further analysis, based on EFA results, for RQ2 were: Fb, ACES, CST, and Bd. The excluded tasks primarily tapped indicators of emotional knowledge/awareness, social problem solving and false beliefs related to theory of mind, and the indicator of general non-verbal cognitive ability.

Next, we conducted CFA analyses with the remaining eight tasks. We tested three competing models: a one-factor model, a two-factor model with correlated factors, and a bifactor model that replaced the factor correlation from the two-factor model with a general cognitive abilities factor (Figure 1). As shown in Table 3, the one-factor CFA model failed the exact fit test, and its TLI was below the good fit criterion of 0.95. Both the two-factor and bifactor models met all criteria for good model fit. Model comparisons revealed that the two-factor model had better relative fit than the one-factor model, BIC difference = 9.48, and the bifactor model, BIC difference = 27.20. The chi square difference test for the one-and two-factor models was significant at an alpha level of 0.05, *χ*^2^ diff (1) = 9.98, *p* = 0.002. The chi square difference test for the two-factor and bifactor models was not significant at an alpha level of 0.05, *χ*^2^ diff (7) = 9.59, *p* = 0.21. This indicates that if the models fit equivalently in the population, then the probability of selecting a sample in which the chi square statistic is 9.59 or greater was 0.21, suggesting that equivalence in the fits of the two-factor and bifactor models cannot be ruled out. In sum, evaluation of global model fit indicated that both the two-factor and bifactor models were consistent with the data, however, direct comparisons of the models favored the more parsimonious two-factor model.

**Figure 2:**
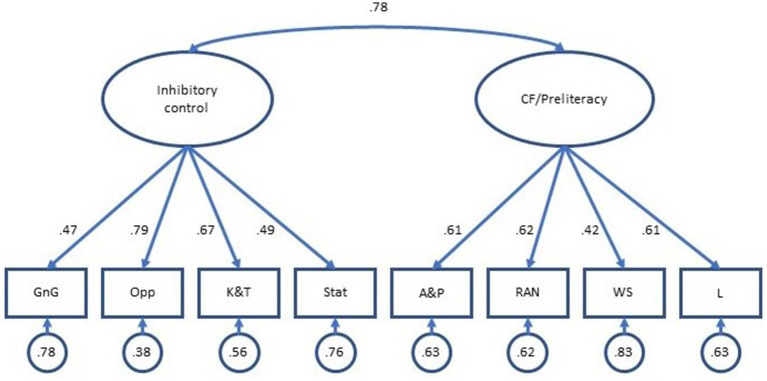
Parameter estimates, two-factor model. Observed variables: GnG, Go/No-go; Opp, Opposites; K&T, Knock and Tap; Stat, Statue; A&P, Apples and Pears; RAN, Rapid Naming; WS, Word Span; and L, Letters.

Parameter estimates from both the two-factor and bifactor models are presented in Table 4. As shown in the table, all standardized factor loading estimates from the two-factor model were positive and ranged from 0.42 to 0.79. As shown in Figure 2, the percentage of variance in child task scores not explained by the factors ranged from 0.38 to 0.83. The correlation between the Inhibitory Control and Cognitive Flexibility/Preliteracy factors was 0.78, 95% CI [0.65, 0.92].

**Table 4:** Factor loadings for the two-factor and bifactor models.

Latent factors	Child task indicators	Estimated factor loadings	
		Standardized	Unstandardized
Two-factor model			
Inhibitory Control	Go/No-go (GnG)	0.46 [0.35, 0.60]	1.50 [0.96, 2.05]
	Knock and Tap (K&T)	0.67 [0.56, 0.78]	4.15 [3.06, 5.24]
	Opposites (Opp)	0.79 [0.67, 0.90]	10.16 [8.07, 12.25]
	Statue (Stat)	0.49 [0.34, 0.65]	3.49 [2.37, 4.62]
Cognitive flexibility/preliteracy	Apples and Pears (A&P)	0.61 [0.48, 0.74]	2.69 [1.92, 3.46]
	Rapid Naming (RAN)	0.62 [0.50, 0.73]	4.20 [3.23, 5.18]
	Word Span (WS)	0.42 [0.32, 0.52]	1.77 [1.30, 2.25]
	Letter Knowledge	0.61 [0.50, 0.71]	5.51 [4.33, 6.69]
Bifactor model			
Cognitive abilities	Go/No-go (GnG)	0.41 [0.22, 0.59]	1.29 [0.72, 1.86]
	Knock and Tap (K&T)	0.62 [0.43, 0.81]	3.87 [2.48, 5.27]
	Opposites	0.78 [0.63, 0.93]	10.05 [7.76, 12.33]
	Statue (Stat)	0.52 [0.37, 0.67]	3.66 [2.57, 4.75]
	Apples and Pears (A&P)	0.50 [0.34, 0.67]	2.20 [1.42, 2.97]
	Rapid Naming (RAN)	0.54 [0.40, 0.67]	3.66 [2.51, 4.82]
	Word Span (WS)	0.35 [0.24, 0.47]	1.51 [1.00, 2.01]
	Letter Knowledge (L)	0.44 [0.31, 0.57]	3.98 [2.78, 5.17]
Inhibitory control	Go/No-go (GnG)	0.40 [−0.17, 0.97]	1.27 [−0.66, 3.21]
	Knock and Tap (K&T)	0.44 [−0.32, 1.20]	2.76 [−1.95, 7.47]
	Opposites (Opp)	0.08 [−0.35, 0.50]	0.97 [−4.60, 6.54]
	Statue (Stat)	−0.07 [−0.36, 0.23]	−0.47 [−2.58, 1.65]
Cognitive flexibility/Preliteracy	Apples and Pears (A&P)	0.29 [−0.06, 0.64]	1.26 [−0.34, 2.85]
	Rapid Naming (RAN)	0.24 [−0.10, 0.57]	1.60 [−0.63, 3.83]
	Word Span (WS)	0.22 [0.01, 0.42]	0.92 [0.04, 1.80]
	Letter Knowledge (L)	0.62 [0.03, 1.21]	5.61 [0.23, 10.98]

Estimates were generated with a robust maximum likelihood estimator. 95% confidence intervals are in brackets. Inhibitory control measures: Go/No-go (GnG), Knock and Tap (K&T), Opposites (Opp), and Statue (Stat). Cognitive flexibility/Preliteracy measures: Apples and Pears (A&P), Rapid Automatized Naming (RAN), Word Span (WS) and Letter Knowledge (L).

**Figure 3:**
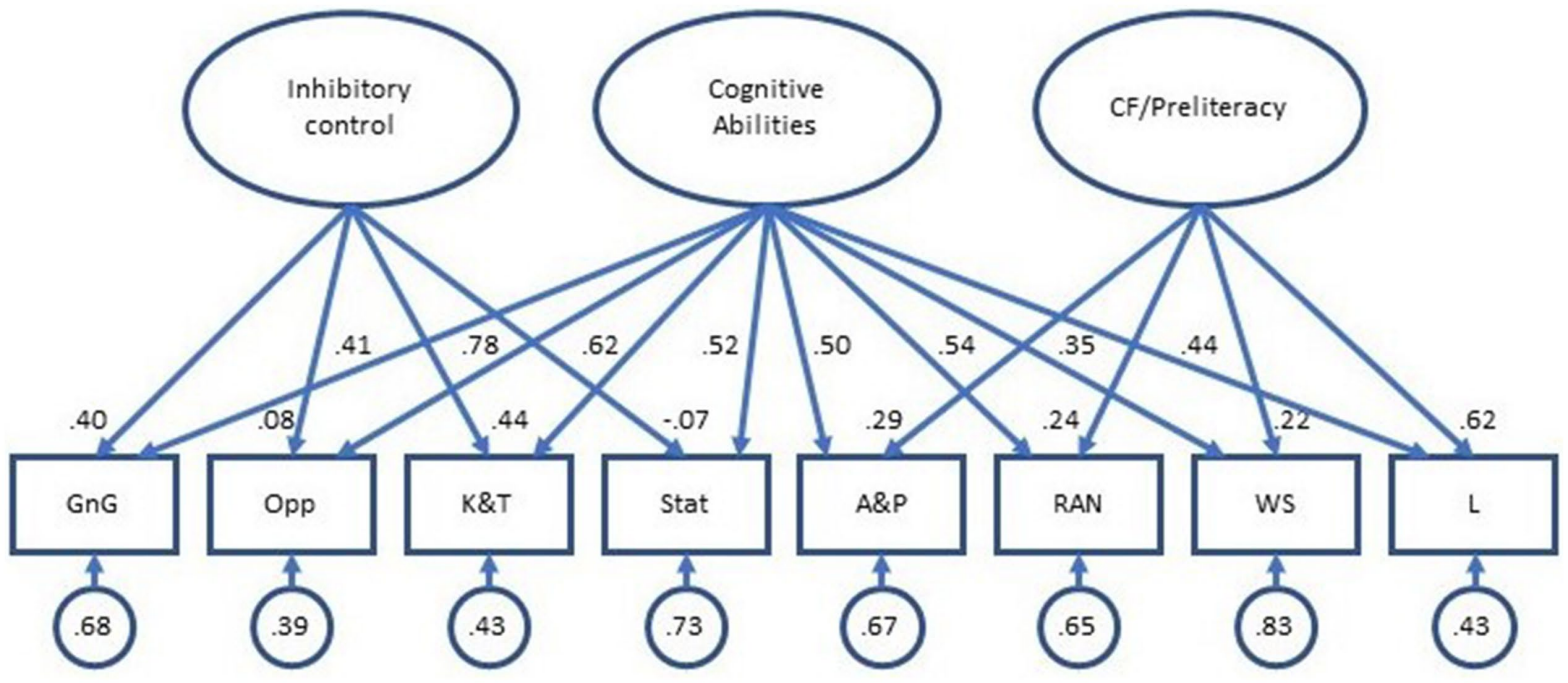
Parameter estimates, bifactor model. Observed variables: GnG, Go/No-go; Opp, Opposites; K&T, Knock and Tap; Stat, Statue; A&P, Apples and Pears; RAN, Rapid Naming; WS, Word Span; and L, Letters.

The standardized factor loading estimates from the bifactor model were more variable. All loadings onto the general Cognitive Ability factor were positive and ranged from 0.35 to 0.78. However, many of the loadings onto the specific Inhibitory Control and Cognitive Flexibility/Preliteracy factors were weaker. Confidence intervals for several child tasks included negative estimates (GnG, K&T, Opp, A&P, and RAN), suggesting that negative factor loadings in the population would be consistent with the sample data in these cases. For one child task (Stat), the factor loading estimate was negative. As shown in Figure 3, the percentage of variance in task scores not explained by the factors ranged from 0.39 to 0.83. In sum, evaluation of both models’ parameter estimates indicated local sources of poor fit for the bifactor model but not for the two-factor model. Specifically, when variance shared by all task indicators was accounted for with a general cognitive abilities factor, the estimated relations between the tasks and latent factors were weaker and more ambiguous.

## 4 Discussion

This study investigated the cognitive abilities (broadly conceptualized) of a sample of four and five-year-old Swedish children, who were attending preschool. Initially, we performed an EFA of a wide range of cognitive abilities, and it was found that the outcome measures of abilities typically viewed as facets of social emotional competence (emotional knowledge/awareness, social problem solving and false beliefs) and the indicator of general non-verbal cognitive ability, did not group (i.e., empirically associate) with measures of preliteracy skills and abilities typically considered as EFs. We proceeded to narrow the scope of the analysis, using the abilities that showed associations in the EFA and were conceptually meaningful. The subsequent CFA analyses indicated that best fitting model out of the three considered was the two-factor model, with one factor involving inhibitory control and one factor involving more complex and high-demanding skills that require more cognitive resources, i.e., working memory, cognitive flexibility, and preliteracy skills. This is in line with previous CFA studies of EF on typically developed kindergartners in Italy (Scionti and Marzocchi, 2021) and Canada (Monette et al., 2015), where a two-factor model with an inhibition factor and a factor presenting working memory and flexibility best fit with the data, and where the two latent factors were differentiated but still significantly correlated.

This study differs from other previous studies, which have included primarily EF tasks, in that we aimed to study cognitive abilities more broadly (i.e., RQ1 with EFA), and investigate how different constructs were associated with one another among preschool aged Swedish children. The choice of tasks was, in part, a reflection of the outcome measures that were included in an intervention study (Eninger et al., 2021), which in turn were based on prior intervention trials of PATHS and the REDI trial. Although several of the original tasks in the EFA were not associated enough to be included in the subsequent CFA, the two tasks measuring preliteracy did. Based on the CFA analyses, a two-factor model in which a factor involving working memory, cognitive flexibility, and preliteracy was identified. This is further in line with previous studies pointing to the close association between language skills and EF in preschool aged children (e.g., Tonér, 2021; Tonér and Gerholm, 2021), and particularly between language and more cognitively demanding EF tasks (Shaul and Schwartz, 2013).

### 4.1 The interplay between EFs and preliteracy

The constructs included in this study captured a broader set of cognitive abilities. One factor included more complex EFs (working memory and cognitive flexibility) as well as preliteracy skills, indicating that these abilities, in this sample, were closely linked already in the preschool years. This is in line with a recent study by Filipe et al. (2023) showing that more complex EFs, as opposed to inhibitory control, are predictive of language ability in preschoolers (see also Shaul and Schwartz, 2013). In our study, we found that complex EFs and preliteracy measures belonged to the same latent factor, and was differentiated from inhibitory control in this sample. The relations between different EF components are likely to change across development, with accelerated development of more complex EFs taking place after the age of 5 years old (Reilly et al., 2022), which points to the importance of conducting longitudinal studies in order to fully understand the intricate dynamics involved. Although this study was cross-sectional and thus not able to speak to the question of development *per se* of these constructs, it does provide a snapshot of the relations between EFs and preliteracy at this age, for this Swedish sample.

Further, given the complex dynamics of development, this study points to the importance of including other cognitive abilities, more than traditional measures of EF, in order to gain a nuanced description of the possible interrelations between cognitive, social emotional, and linguistic development. Including broader measures of development can also be motivated by more recent conceptions of EF, where the experiences and learning of a child prompts the use of EF’s, for example that developing EF’s under more adaptive behaviors (Doebel, 2020) or that development of EF can be conceptualized as a response to environmental cues (Munakata and Michaelson, 2021). Indeed, existing theories about the development of EF support the idea that less demanding skills, like inhibitory control, develop early in life, and that more complex skills are considered to build on simple skills and, thus, develop later (e.g., Best and Miller, 2010; Bornstein et al., 2006; Garon et al., 2008; Ibbotson, 2023).

### 4.2 Differentiation of EFs – the timeline of unfolding

In the research literature about EFs, there is an ongoing discussion as to when in development, the components of EF differentiate from a single generic regulatory factor (i.e., Bailey and Jones, 2019; Blair, 2016) to a two, and later, three-factor structure, as has been identified in young adults (Karr et al., 2018; Miyake et al., 2000, 2012). Thus, indicating a movement from greater unity in EF functioning earlier in development to greater diversity later in development in functions that remain interdependent and closely aligned in everyday life tasks and experiences (Baggetta and Alexander, 2016). Our results, i.e., a two-factor model reflecting cognitive abilities in this sample of four-to-five-year-old children, are in line with some previous studies (e.g., Karr et al., 2018; Michel and Bimmüller, 2023; Monette et al., 2015; Scionti and Marzocci, 2021) regarding the timing of differentiation, although there is some inconsistency in the field with other studies pointing to only one factor, however for 3–6 years old children (e.g., Fuhs and Day, 2011; Wiebe et al., 2008). Explanations for this inconsistency may lie in the range of tasks included across studies; it is well known that the number of indicators of a construct and the specific characteristics of a task can influence associations between tasks, and thus the subsequent factor structure obtained (e.g., Miller et al., 2012; Karr et al., 2018).

In the present study, a bifactor solution, in which there is a common underlying factor explaining some of the variance in functions along with more clearly differentiated factors, was not entirely supported by the data. In that there was some diversity in constructs measured by our tasks, it may have made it more difficult to find one common underlying factor. This is further in line with the reasoning by Karr et al. (2018) in their re-analysis of latent factor models, regarding the difficulty in finding model convergence for bifactor models, in general. The rather substantial correlation between our factors also indicates that there is a considerable overlap in constructs, so it is not surprising that, depending on which tasks are included, the content of factors may vary between studies. Still, the two-factor solution was found to be both parsimonious and well-fitting the present study data, and overall our results are in line with the view that EF consists of related but separable components (Miyake et al., 2000), even in young children.

### 4.3 EF and the Swedish context

An interesting point for discussion, beyond the proposed structure of cognitive abilities, is perhaps what it is that affects the development of these abilities during the preschool years. Although this study was cross-sectional and therefore cannot inform us of the developmental or causal aspects of processes connected with the examined study constructs, the cultural context differs in some regards from of the extant studies in this field. A vast majority of studies of cognitive abilities, including executive functions and SEC, in young children have been performed in the US. As already mentioned, the present study was conducted in a Swedish preschool context, which is influenced by the Nordic welfare model, which may be assumed to differ somewhat from the context of previous studies. In general, the influence of preschool context on early child development is understudied, but studies have shown that there are differences across countries in the organization and goals of ECEC, as well as in the education of preschool teachers, and their practices in the classroom (Coelho et al., 2021). That being said, it may not be assumed that what may be true of a specific cultural context also holds at an individual or family level. In addition, Sweden is a multi-cultural society with many Swedes that have diverse immigration experiences, including children who are being raised with a diversity of norms and values, for instance regarding parenting and point of view as regards to early childhood education and care. Indeed, the contextual aspects of preschools and families presented above may be important to consider in studies of cognitive abilities in young children attending preschools. These factors were not assessed in this study and represent a promising direction for future research.

### 4.4 Limitations, strengths, and future directions

There are several study limitations that should be considered in light of the main study findings. As noted, these include the use of cross-sectional study design. Although the CFA models posited in this study were informed by a consideration of the development of EF from prior empirical studies and theory, the study design used here cannot speak to developmental change, sequence of processes at work, or causality among the measured constructs, and captures only a particular timepoint in the development of this sample.

The sample itself ranged in age and included four and five-year-old children, and were part of a wider study that included the test of an SEL intervention. Thus, there are very likely to be several selection factors operating to make this sample unique (e.g., schools interested in participating in a time limited intervention trial of an SEL intervention), and possibly not representative of Swedish children at this age in general. The study was also conducted in preschools in a large urban Swedish city and generalizations to children living in other parts of Sweden are also limited and should be examined by further research.

The array of tasks used in this study, while a strength in their diversity, also pose a possible limitation in that the use of other indicators or tasks of the same construct could result in drawing different conclusions about the overall organization of the examined cognitive abilities. In addition, the more demanding tasks were challenging for some children, who were unable or unwilling to complete the tasks. Failure to complete a task can be related to many causes, such as disinterest or inability to muster attention, as well as difficulty understanding the task, or fatigue. Moreover, analyses of some of these tasks suggested modest to low reliabilities. Therefore, it is useful to conduct further research with other indicators of the same constructs to confirm or qualify the present study findings. All indicators of cognitive abilities were from the perspective of the child’s performance in these tasks, but other indicators from the viewpoint of teachers and parents of children’s abilities could be an important complement to the type of data examined in this study. Other indicators of context of the children’s development, like preschool, classroom, home, neighborhood environment/climate, were not comprehensively examined in this study and would provide great insight in future studies that examine children’s cognitive abilities within key developmental contexts. In addition, other indicators involving, e.g., early self-regulation, attention, and self-talk could be of interest to include in future studies in order to further our understanding of the complex interrelations between EF, SEC, literacy and school readiness. These limitations should also be viewed in light of the several strengths and contributions of this study. There are several scientific/technical study strengths including the use of structural equation modeling CFA measurement models that were empirically and theoretically motivated, combined with a competitive testing of several plausible measurement models. Whereas a score yielded by any single measure of cognitive ability will be error-laden and to some degree unreliable, using scores from multiple measures to model cognitive abilities as latent variables allowed us to correct these latent variables for sources of measurement error (Little, 2024). Of course, further models could be possible to evaluate, however, the models tested in the present study represented a wide diversity of theoretical perspectives and extant empirical findings. Moreover, the use of several widely used cognitive ability tasks that evidenced sound psychometric properties (in this study and prior studies) was also a clear study strength. In addition, the tasks were administered in a standardized way and in a real-life context of children’s development, namely in children’s preschools. A further, and major, strength was that this study fills an important gap in the Swedish research literature, as cognitive abilities in early childhood has not been extensively studied in the past.

The decision to retain study constructs for RQ2 was based on empirical grounds (i.e., EFA results, see Table 3) as well as based on the CASEL model, a theory of social emotional competence, which was a guiding conceptual framework for this study (Collaborative for Academic, Social, and Emotional Learning [CASEL], 2024; Lawson et al., 2019; Weissberg et al., 2015). Individual competencies within a CASEL domain would likely be connected to one another in a more profound way relative to competencies spread across domains (Collaborative for Academic, Social, and Emotional Learning [CASEL], 2024; Weissberg et al., 2015). The present study results indicated that competencies within the CASEL self-management domain (i.e., inhibitory control measured by GnG, K&T, Opp, Stat, cognitive flexibility and working memory measured by A&P and WS) were connected to one another and this made conceptual sense from a CASEL standpoint (all indicators conceptually fitting in a self-management domain). The SE skills not retained for RQ2, could be viewed as single indicators spread across three different CASEL domains. In other words, emotional knowledge could be seen as a single indicator within the domain of self-awareness (measured by ACES), social problem solving as a single indicator of relationship skills (measured by CST), and theory of mind (false belief) as a single indicator of social awareness (as measured by Fb). A larger sample size and more indicators per CASEL domain, than were the case in this study, could yield different substantive conclusions about how the measured social emotional competence domains relate to one another. Gaining deeper insight into the structure of social emotional development can only be achieved through intervention and longitudinal research (e.g., Whittaker et al., 2024), and the present study only offers a snap shot in time of a limited set of indicators. Future research would also benefit from an even greater theory driven measurement of social emotional competence with multiple indicators across key domains and with different theories about social emotional competence and learning considered in terms of measurement (Cipriano et al., 2023). Future research would also benefit from a deeper consideration of the particulars and possible synergies of task demands, the complexity and novelty of multiple tasks when given as a complete assessment from the standpoint of young children.

Finally, this study has theoretical contributions to the scholarly debate on EF structure during childhood, and provides an empirical illustration of the close association between complex EFs and preliteracy skills in this present sample, which provides a sound empirical rationale for the further development of SEL interventions that consider young children’s preliteracy skills. Indeed, the inclusion of cognitive abilities that are not typically considered part of the wider conceptual umbrella term EF, like other SECs, and indicators of preliteracy in a preschool aged sample, remains relatively unique within the wide child development literature. The REDI trial conducted by Bierman et al. (2008a,b) is an exception in that it was a watershed study which demonstrated the potential of systematically pairing a focus on preliteracy with a well-developed evidence based early SEL intervention (i.e., PATHS, Domitrovich et al., 2007). The REDI trial (Bierman et al., 2008a,b) and other studies show the potential of this approach.

From a practical standpoint, there are several reasons to motivate why it is desirable to support children in their social emotional, cognitive and linguistic development. First, the connection of SEL to academic readiness for primary school is often discussed when there is a consideration of whether to take on SEL intervention within preschools. However, the present study exhibits an example of the interconnectedness of SEL indicators like EF (as viewed from a CASEL theoretical model) with preliteracy skills. This adds a potent empirical illustration of why a unified intervention approach that includes core effective components of SEL interventions paired with a strategic emphasis on supporting children’s preliteracy skills, is a valuable investment for preschools and society more generally. Furthermore, given the setting of the present study, more attention should be placed on the possible impact of contextual factors on the development of cognitive abilities. Values regarding child rearing and contextual aspects of Swedish preschools would be important areas to consider in future research in order to gain additional insight into the development of Swedish children’s cognitive abilities, including abilities that support school readiness.

## 5 Conclusion

This study examined a wide array of cognitive abilities that included abilities connected with social emotional competence, as well as executive functions and preliteracy skills. By taking such a wide approach, we found some evidence to connect complex EFs with preliteracy skills in this sample of Swedish preschool aged children. Using CFA, we demonstrated a two-factor model with one inhibitory control factor and one factor including working memory, cognitive flexibility and preliteracy skills. These two latent factors were significantly associated with each other, which also supports the view of unity and diversity of EFs. Thus, the findings of this study contribute to new information about the structure of cognitive abilities in young children, also in an understudied (for this wide set of constructs) cultural and preschool context.

## Data Availability

The datasets presented in this article are not readily available because the ethical review for this study prohibits public posting of the datasets that are the basis of this article. In cases of meta-analysis or confirmation of published study results, de-identified data requests can be made. Requests should be made by qualified researchers (e.g., Ph.D.) along with ethical permission under Swedish law regarding secondary data analysis. Requests to access the datasets should be directed to PATHS_PW.Project@psychology.su.se.
