# Supplementary material for: Cognitive abilities in a sample of young Swedish children
Source: Front Psychol. 2024 Dec 3;15:1398398. doi: 10.3389/fpsyg.2024.1398398 (PMC11649402; doi:10.3389/fpsyg.2024.1398398)
Supplement: Supplementary file 1 [file Table_1.DOCX]

| **Scale**  **Name** | False belief (Fb) | ACES | CST competent | Apples and Pears (A&P) | RAN | Word Span (WS) | Letter Knowledge  (L) | Go-Nogo (GnG) | Block Design (Bd) | Knock and Tap  (K&T) | Opposites  (Opp) | Statue  (Stat) |  |
| --- | --- | --- | --- | --- | --- | --- | --- | --- | --- | --- | --- | --- | --- |
| False Belief (Fb) |  | **.129^*^** | 0.072 | **.255^**^** | **.295^**^** | **.187^**^** | **.308^**^** | **.209^**^** | 0.101 | **.213^**^** | **.271^**^** | **.178^*^** |  |
| ACES |  |  | 0.047 | **.135^*^** | **.173^**^** | -0.043 | **.144^*^** | 0.106 | **.131^*^** | **.142^*^** | 0.052 | **.184^**^** |  |
| CST competent |  |  |  | 0.000 | **.147^*^** | **.134^*^** | 0.092 | 0.116 | **.131^*^** | 0.114 | 0.084 | -0.007 |  |
| Apples and Pears (A&P) |  |  |  |  | **.357^**^** | **.240^**^** | **.390^**^** | **.226^**^** | **.176^**^** | **.244^**^** | **.386^**^** | **.264^**^** |  |
| RAN |  |  |  |  |  | **.186^**^** | **.384^**^** | **.214^**^** | **.239^**^** | **.327^**^** | **.394^**^** | **.262^**^** |  |
| Word span (WS) |  |  |  |  |  |  | **.300^**^** | 0.120 | 0.053 | **.254^**^** | **.246^**^** | **.194^**^** |  |
| Letter Knowledge (L) |  |  |  |  |  |  |  | 0.088 | **.133^*^** | **.287^**^** | **.343^**^** | **.184^**^** |  |
| Go-Nogo (GnG) |  |  |  |  |  |  |  |  | **.208^**^** | **.378^**^** | **.303^**^** | **.140^*^** |  |
| Block Design (Bd) |  |  |  |  |  |  |  |  |  | **.156^*^** | **.189^**^** | 0.097 |  |
| Knock and Tap (K&T) |  |  |  |  |  |  |  |  |  |  | **.468^**^** | **.254^**^** |  |
| Opposites (Opp) |  |  |  |  |  |  |  |  |  |  |  | **.372^**^** |  |
| *Note*. Scale names are: False belief (Fb), Assessment of children’s emotional skills (ACES), Challenging situations task (CST) – competent response scale score, Apples and Pears (A&P), Rapid Automatized Naming (RAN), Word Span (WS), Letter Knowledge (L), Go/No-go (GnG), Block design (Bd), Knock and Tap (K&T) Opposites (Opp) Statue (Stat); * = Pearson correlation coefficient was significant at the .05 level (2-tailed) and ** at the .01 level (2-tailed). Significant correlations are bolded in the table. All significant correlations were positive and ranged from .129 to .468 | | | | | | | | | | | | | |

**Supplementary Table 1**. Pearson bi-variate correlations for study scales
